# The Lag -Effects of Air Pollutants and Meteorological Factors on COVID-19 Infection Transmission and Severity: Using Machine Learning Techniques

**DOI:** 10.34172/jrhs.2024.157

**Published:** 2024-07-31

**Authors:** Nadia Mohammadi Dashtaki, Alireza Mirahmadizadeh, Mohammad Fararouei, Reza Mohammadi Dashtaki, Mohammad Hoseini, Mohammad Reza Nayeb

**Affiliations:** ^1^Student Research Committee, Shiraz University of Medical Sciences, Shiraz, Iran; ^2^Non-communicable Diseases Research Center, Shiraz University of Medical Sciences, Shiraz, Iran; ^3^AIDS/HIV Research Center, School of Public Health, Shiraz University of Medical Sciences, Shiraz, Iran; ^4^Department of Chemistry, Isfahan University of Technology, Isfahan, Iran; ^5^Department of Environmental Health Engineering, School of Health, Shiraz University of Medical Sciences, Shiraz, Iran

**Keywords:** Air pollutants, Meteorological factors, COVID-19, Machine learning, Time factors

## Abstract

**Background:** Exposure to air pollution is a major health problem worldwide. This study aimed to investigate the effect of the level of air pollutants and meteorological parameters with their related lag time on the transmission and severity of coronavirus disease 19 (COVID-19) using machine learning (ML) techniques in Shiraz, Iran.

**Study Design:** An ecological study.

**Methods:** In this ecological research, three main ML techniques, including decision trees, random forest, and extreme gradient boosting (XGBoost), have been applied to correlate meteorological parameters and air pollutants with infection transmission, hospitalization, and death due to COVID-19 from 1 October 2020 to 1 March 2022. These parameters and pollutants included particulate matter (PM2), sulfur dioxide (SO_2_ ), nitrogen dioxide (NO_2_ ), nitric oxide (NO), ozone (O_3_ ), carbon monoxide (CO), temperature (T), relative humidity (RH), dew point (DP), air pressure (AP), and wind speed (WS).

**Results:** Based on the three ML techniques, NO_2_ (lag 5 day), CO (lag 4), and T (lag 25) were the most important environmental features affecting the spread of COVID-19 infection. In addition, the most important features contributing to hospitalization due to COVID-19 included RH (lag 28), T (lag 11), and O_3_ (lag 10). After adjusting for the number of infections, the most important features affecting the number of deaths caused by COVID-19 were NO_2_ (lag 20), O_3_ (lag 22), and NO (lag 23).

**Conclusion:** Our findings suggested that epidemics caused by COVID-19 and (possibly) similarly viral transmitted infections, including flu, air pollutants, and meteorological parameters, can be used to predict their burden on the community and health system. In addition, meteorological and air quality data should be included in preventive measures.

## Background

 Exposure to air pollution is a major global health problem. According to a report from the World Health Organization (WHO), more than 90% of the world’s population lives in cities with low air quality, and the concentration of air pollutants in these cities exceeds the air quality standards of the WHO. It is also estimated that each year, exposure to air pollution causes 7 million premature deaths and the loss of millions more years of healthy life.^1^ Exposure to air pollutants such as carbon monoxide (CO), nitrogen dioxide (NO_2_), sulfur dioxide (SO_2_), ozone (O_3_), PM2.5, and PM10 are known as the major causes of health conditions such as respiratory and cardiovascular diseases, premature death, and diabetes.^2,3^. The occurrence of such chronic diseases is associated with an increased risk of mortality among people with COVID-19.^4^

 In December 2019, coronavirus disease 2019 (COVID-19), caused by severe acute respiratory syndrome-related coronavirus 2 (SARS-CoV-2), was first reported in Wuhan, China, and shortly became a global pandemic.^5,6^ The severity and mortality of COVID-19 are associated with several factors, such as some comorbidities, sociodemographic factors, living conditions, and environmental factors, including pollutants.^7,8^ In this regard, several researchers hypothesized that the disease could spread more rapidly due to the occurrence of various environmental conditions.^9,10^

 Studies suggest that exposure to air pollutants increases the risk of transmission of viral infections.^11,12^ For example, the transmission of SARS-CoV-2 infection is associated with certain levels of air pollutants, air temperature (T), and relative humidity (RH).^10,1^ Several studies suggest that certain infectious agents, including coronaviruses, may survive longer in polluted air^14,15^ and that exposure to air pollutants, especially particulate matter (PM) and toxic gases, can weaken the body’s respiratory defense by damaging the lining of the respiratory tract, making it easier for viruses to penetrate and form infection.^15,16^ In addition, air pollution can trigger inflammation in the respiratory system and suppress the function of the immune system, making individuals more susceptible to severe infections. For instance, evidence indicates that exposure to air pollutants may increase the risk of hospitalization and death among individuals infected by COVID-19.^12,15^ Making the subject more complicated, it seems that meteorological factors not only affect the level of air pollutants but also may alter the effect of pollutants on the body’s functions.^17,18^

 Although several studies have been conducted on the role of environmental factors in the incidence and mortality of COVID-19 infection during the 2019 pandemic, considering the long list of meteorological factors and air pollutants, very few have spotted the light on the time interval between changes in the levels of air pollutants and meteorological parameters and the incidence of COVID-19 infection and its severity (i.e., hospitalization and death), in one modeling framework.^19^

 Machine learning (ML) methods have been extensively used to detect and predict various diseases.^20^ Therefore, the current study was conducted to define the relative importance of meteorological and pollution parameters in predicting the infection and mortality of COVID-19 using ML techniquesand investigate the lag time effects of air quality factors on the outcome indexes. The selected air parameters included T, RH, dew point (DP), air pressure (AP), and wind speed (WS), as well as the concentrations of PM2, SO_2_, NO_2_, NO, O_3_, and CO.

## Methods

###  Setting 

 This ecological study was conducted from October 1, 2020, to March 1, 2022, in Shiraz. This period was selected because the least changes were observed in applying the major control strategies, including vaccination, diagnosis, community restrictions, and the like. Shiraz is the capital of Fars Province, located in the south of Iran. Its population size is about 1 955 500 people, making Shiraz the fourth largest and most populous city in Iran. The average annual T of the city is about 17.8 °C, with a maximum T of 43.2 °C in July and a minimum T of below-freezing in January. The average annual rainfall is about 283.9 mm, and the average height of this city is 1486 meters above sea level.

###  Air pollution data

 The data on air pollutants, including CO, O_3_, NO_2_, SO_2_, PM2.5, and NO, were collected from the Environmental Organization of Fars Province. The organization measures the selected pollutants on an hourly basis. After scanning the data, the outliers and missing values of various pollutants have been defined and imputed by the random forest (RF) method. Then, the average concentration over 24 hours was calculated for the pollutants. The average concentration over 24 hours was used in this study.

###  Meteorological parameters 

 The meteorological data from October 1, 2020, to March 1, 2022, which was utilized in the present study included AP, T, DT, RH, and WS. The data are available at https://www.wunderground.com.

###  Coronavirus disease 19 data

 The required COVID-19 data (i.e., the number of positive tests based on polymerase chain reaction tests and the number of hospitalizations and deaths due to COVID-19 daily) were obtained from the COVID-DASHBOARD and MCMC (Medical Care Monitoring Center) databases of Shiraz University of Medical Sciences. The reported COVID-19 data are being regularly updated daily.

###  Statistical analysis 

 After checking the normality assumption, the Pearson correlation coefficient was used to measure the lag time effect of independent variables, namely, environmental (O_3_, NO_2_, SO_2_, PM2.5, NO, and CO) and meteorological (AP, T, DP, RH, WS) parameters on the number of cases, number of hospitalizations, and number of deaths due to COVID-19. A relationship was considered statistically significant at *P* < 0.05. Statistical analysis was performed using the STATA software, version 17.

 Lag times with the strongest correlation were selected to be included in the ML procedure to define the relative importance of the parameters. The relative importance of the air parameters is defined via conducting an ML approach (a well-known approach playing a crucial role in the epidemiology of diseases by providing more accuracy in description, prediction, and decision-making in advanced epidemiological studies).^21^ Three modeling methods (i.e., decision tree [DT], RF, and extreme gradient boosting [XGBoost]) were employed to define and compare three lists of the relative importance of the study parameters in the incidence and severity of COVID-19 infection.^22,23^ In general, DTs are basic models that can overfit despite being widely used. RF is an ensemble method that reduces overfitting by averaging predictions, and XGBoost is a powerful gradient-boosting algorithm that sequentially improves model performance by correcting errors. Each algorithm has its strengths and is suitable for different aspects of our study questions. All methods are utilized to define the relative importance of the study parameters and the majority voting index.

 Among several measures of model performance (e.g., R-squared, mean absolute error, mean squared error, and root mean square error) available in similar areas of research,^24^ R^2^ was used to compare the performance of the models. This is because it seems that R^2^ is a more informative and comparative metric.^25^ The models were trained and validated using the Python software, version 3.8.

###  Machine learning approach

 In this study, feature selection techniques based on ML methods were employed to identify the various environmental and meteorology parameters that affect the number of cases, hospitalizations, and deaths due to COVID-19. Assuming the usual time lag from virus transmission to apparent infection (Max. = 14 days), infection to hospitalization (Max. = 10 days), and death (Max. = 10 days),^26^ the lag times of the effects of daily air pollutants and meteorological parameters on the COVID-19 infection and death were set between 0 and 30 days. Moreover, considering the important effect of the number of positive cases diagnosed on the number of hospitalizations and the number of deaths, this variable was entered into the model.

**Figure 1:**
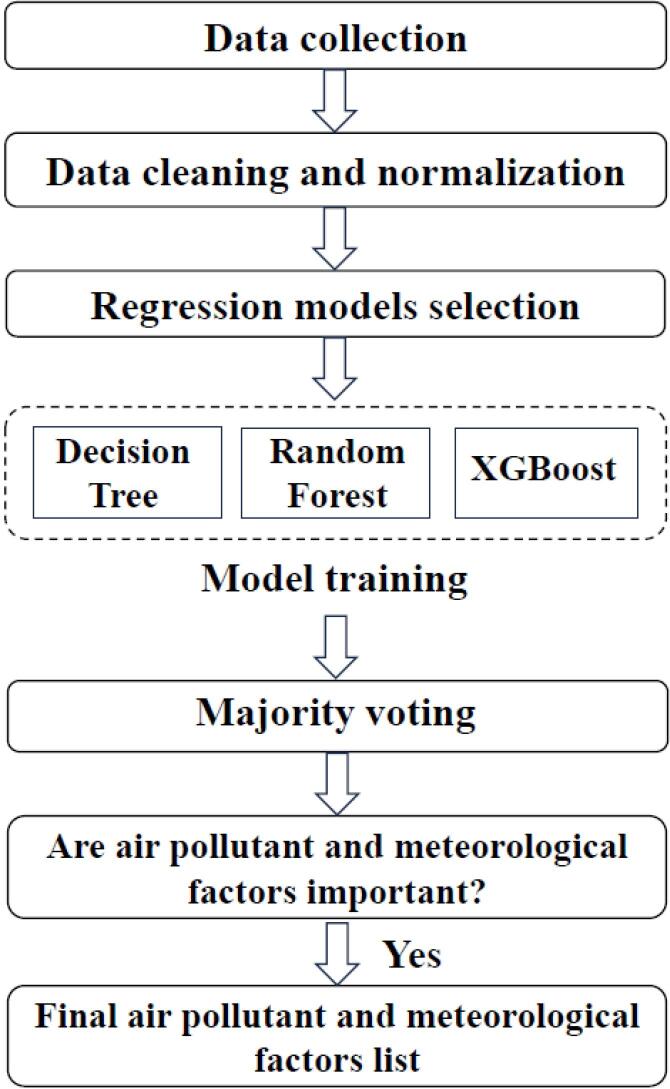
Flow chart for machine learning workflow. *Note*. XGBoost: Extreme gradient boosting

 The operational flow of the ML algorithm is depicted in Figure 1.

1. The datasets of air pollutants (PM2.5, CO, NO_2_, NO, SO_2_, and O_3_) and meteorological parameters (AP, air T, DP, RH, and WS) were used as predictors, and the number of cases, number of hospitalizations, and number of deaths due to COVID-19 were employed as response variables.
2. To preprocess the dataset, the missing values of various pollutants were imputed by the RF method. The imputed dataset was split into 80% as train data and 20% as test data.
3. In this study, several ML techniques (i.e., DTs, RF, and XGBoost) were utilized to analyze the data via modeling and computing the feature’s importance and lag time of their effects. These techniques are described in more detail in the following sections:

####  Decision tree

 DT is a non-linear algorithm. It represents the parameters by a node in the tree, and the values of these parameters are represented by the respective branches of the node. The DT performs the division of input based on the values of various parameters. A DT classifier’s design is influenced by the way the tree is structured, the feature subsets used at each internal node, and the decision criteria utilized at each node. Some of the criteria for the design of the tree structure include error rates, the number of nodes in the tree, and information gain. Entropy and information-based techniques are generally employed as a decision rule at each node, whereas branch and bound techniques and greedy algorithms are applied for feature subset selection.^19^

####  Random forest

 The RF constitutes a supervised ensemble learning methodology that predicts based on DTs. This approach involves the prediction of multiple trees, each independently trained, and then the final result is averaging the values. Samples from the dataset equal to the number of trees needed to build the RF are taken, and a tree is grown by selecting the best split among all the input variables at each node. The process of making predictions is performed by aggregating all of the sample trees’ predictions. It is highly flexible and fast; it can be used for both classification and regression. The majority voting among predictions and the average of predictions are utilized for classification and regression, respectively.^19,27^

####  Extreme gradient boosting

 XGBoost is a successful ML method based on a gradient-boosting algorithm. It has better control over overfitting by using more regularized model formalization in comparison to many other algorithms. A grid search on hyperparameters with 10-fold cross-validation was performed to find the best model based on R^2^ metrics.^27^

4. To train the model using the ML techniques, first, the tuning parameters for each model are chosen, and then resampling is performed using the cross-validation method.
5. The importance of the feature is computed based on the model statistic. A feature is considered important if a reduction in the model statistic is observed when that feature is added to the model. For DT, RF, and XGBoost, the MSE is employed as the model statistic. The performance of each ML model was measured and compared using *R*^2^. ^24,28^
6. The importance of the selected final factors was determined by majority voting with intermediate-level fusion approaches ( ≥ 66%) to integrate the results of the three mentioned ML techniques.^29^

In majority voting, a group of diverse ML models is trained on the same dataset, each employing unique algorithms or techniques. When it is time to make a prediction, each model casts its vote for the outcome, and the final prediction is determined by the majority’s decision. It is a useful technique for improving the accuracy and robustness of the final prediction, particularly when the individual models have different strengths and weaknesses, and their combination can lead to better overall performance.

## Results

 In total, 235 766 cases, 29 894 hospitalizations, and 3,229 deaths due to COVID-19 were registered with the COVID-DASHBOARD and MCMC registry in Shiraz during the study period (515 days). Correlations between daily air pollutants and meteorological factors with the number of cases, number of hospitalizations, and number of deaths due to COVID-19 are provided in Table 1.

**Table 1:** The highest correlation between daily air pollutants and meteorological factors with the number of cases, number of hospitalizations, and number of deaths due to COVID-19 at different lag times (0–30 days)

**Variables**	**Number of cases**			**Hospitalizations**			**Deaths**		
	**Lag (day)**	***r***	***P *** **value**	**Lag (day)**	***r***	***P *** **value**	**Lag (day)**	***r***	***P *** **value**
NO_2_ (ppb)	8	0.63	0.001	0	0.39	0.001	20	0.73	0.001
O_3_ (ppb)	6	0.46	0.001	2	0.18	0.001	23	0.60	0.001
NO (ppb)	27	-0.31	0.001	2	-0.20	0.001	22	-0.42	0.001
SO_2_ (ppb)	2	-0.05	0.260	29	-0.09	0.041	1	-0.08	0.070
CO (ppm)	30	-0.24	0.001	2	-0.24	0.001	27	0.11	0.010
PM2.5 (µg/m−3)	10	0.26	0.001	6	0.20	0.001	13	0.30	0.001
Temperature (°F)	19	0.28	0.001	1	0.12	0.006	30	0.35	0.001
Humidity (%)	26	-0.25	0.001	12	-0.09	0.040	30	-0.32	0.001
Wind speed (mph)	16	0.25	0.001	16	0.29	0.001	28	0.18	0.001
Dew point (°F)	0	0.35	0.001	0	0.20	0.001	0	0.34	0.001
Pressure (in)	30	-0.33	0.001	28	-0.28	0.001	29	-0.23	0.001
Number of cases	-	-	-	0	0.79	0.001	10	0.74	0.001

*Note*. COVID-19: Coronavirus disease 19; NO_2_: Nitrogen dioxide; O_3_: Ozone; NO: Nitric oxide; SO_2_: Sulfur dioxide; CO: Carbon monoxide; PM: Particulate matter.

 Based on the results, the number of cases of COVID-19 had a positive and significant correlation with O_3_, NO_2_, PM2.5, T, DP, and WS. On the other hand, a negative correlation was observed between the number of cases of COVID-19 and NO, CO, humidity, and pressure. In addition, the number of hospitalizations due to COVID-19 had a positive correlation with the detected number of cases of COVID-19, O_3_, NO_2_, PM2.5, T, DP, and WS, while there was a negative correlation with NO, CO, SO_2_, humidity, and pressure. The number of deaths due to COVID-19 had a positive correlation with the detected number of cases of COVID-19, O_3_, NO_2_, CO, PM2.5, T, WS, and DP, while there was a negative correlation with NO, humidity, and pressure. For daily NO_2_, the highest correlation with the number of COVID-19 cases was found at lag 8 day (*r* = 0.63), lag 0 day with hospitalizations due to COVID-19 (*r* = 0.39), and lag 20 day with deaths due to COVID-19 (*r*= 0.73).

 The importance of each air pollutant and meteorological factor, as computed by the various ML techniques, is presented in Tables 2–4, demonstrating the performance of three ML methods using R^2^ results.

**Table 2:** The relative importance of air pollutants and meteorological factors on COVID-19 infection transmission based on machine learning techniques

**Decision tree, *****R***^2^**=0.56**		**Random forest, *****R***^2^**=0.64**		**XGBoost, *****R***^2^**=0.73 **	
**Variables**	**Importance**	**Variables**	**Importance**	**Variables**	**Importance**
NO_2_ (Lag 5 day)	100.00	NO_2_ (Lag 5 day)	100.00	NO_2 _(Lag 5 day)	100.00
Temperature (Lag 25 days)	39.39	CO (Lag 4 day)	26.49	CO (Lag 4 day)	32.59
CO (Lag 4 day)	26.67	Temperature (Lag 25 day)	25.51	Temperature (Lag 25 days)	29.06
O_3_ (Lag 13 day)	25.56	O3 (Lag 13 day)	22.17	PM2.5 (Lag 15 day)	19.41
PM2.5 (Lag 15 day)	22.38	PM2.5 (Lag 15 day)	20.19	Pressure (Lag 22 day)	9.43
SO_2_ (Lag 0 day)	12.97	Humidity (Lag 27 day)	17.70	Dew point (Lag 15 day)	7.50
NO (Lag 26 day)	11.88	Wind speed (Lag 13 day)	9.02	O_3_ (Lag 22 day)	7.45
Humidity (Lag 7 day)	9.09	NO (Lag 26 day)	9.00	Wind speed (Lag 30 day)	7.20
Wind speed (Lag 19 day)	5.12	Dew point (Lag 13 day)	8.94	SO2 (Lag 4 day)	7.12
Pressure (Lag 20 day)	3.46	Pressure (Lag 30 day)	1.88	Humidity (Lag 26 day)	6.79
Dew point (Lag 21 day)	3.12	SO_2_ (Lag 0 day)	6.14	NO (Lag 2 day)	2.03

*Note*. COVID-19: Coronavirus disease 19; NO_2_: Nitrogen dioxide; O_3_: Ozone; NO: Nitric oxide; SO_2_: Sulfur dioxide; CO: Carbon monoxide; PM: Particulate matter; XGBoost: Extreme gradient boosting.

**Table 3:** The relative importance of air pollutants and meteorological factors on hospitalization due to COVID-19 based on machine learning techniques

**Decision tree, *****R***^2^**=0.68**		**Random forest, *****R***^2^**=0.79**		**XGBoost, *****R***^2^**=0.87 **	
**Variables**	**Importance**	**Variables**	**Importance**	**Variables**	**Importance**
Number of cases (Lag 0 day)	100.00	Number of cases (Lag 0 day)	100.00	Number of cases (Lag 0 day)	100.00
Humidity (Lag 28 day)	39.39	Temperature (Lag 11 day)	11.67	Humidity (Lag 28 day)	13.16
Temperature (Lag 11 day)	17.37	Humidity (Lag 28 day)	11.40	Temperature (Lag 11 day)	7.40
O_3_ (Lag 10 day)	7.49	O_3_ (Lag 10 day)	11.30	Dew point (Lag 18 day)	4.92
CO (Lag 10 day)	7.16	SO_2_ (Lag 29 day)	4.60	O_3_ (Lag 10 day)	4.87
PM2.5 (Lag 0 day)	5.98	NO_2_ (Lag 0 day)	4.46	CO (Lag 12 day)	4.59
Wind speed (Lag 16 day)	3.88	PM2.5 (Lag 18 day)	4.45	PM2.5 (Lag 0 day)	3.07
NO (Lag 10 day)	3.65	Wind speed (Lag 16 day)	4.23	NO_2_ (Lag 5 day)	2.75
SO_2_ (Lag 29 day)	1.72	Dew point (Lag 18 day)	4.23	NO (Lag 5 day)	1.94
Dew point (Lag 22 day)	1.23	CO (Lag 12 day)	4.03	Pressure (Lag 28 day)	1.63
NO_2_ (Lag 6 day)	1.12	NO (Lag 11 day)	3.42	Wind speed (Lag 16 day)	1.62
Pressure (Lag 12 day)	0.24	Pressure (Lag 29 day)	0.71	SO_2_ (Lag 1 day)	1.27

*Note*. COVID-19: Coronavirus disease 19; NO_2_: Nitrogen dioxide; O_3_: Ozone; NO: Nitric oxide; SO_2_: Sulfur dioxide; CO: Carbon monoxide; PM: Particulate matter; XGBoost: Extreme gradient boosting.

**Table 4:** The Relative importance of air pollutants and meteorological factors on mortality due to COVID-19 based on machine learning techniques

**Decision tree, *****R***^2^**=0.53**		**Random forest, *****R***^2^**=0.63**		**XGBoost, *****R***^2^**=0.72 **	
**Variables**	**Importance**	**Variables**	**Importance**	**Variables**	**Importance**
Number of cases (Lag 11 days)	100.00	Number of cases (Lag 11 days)	100.00	Number of cases (Lag 11 days)	100.00
O_3_ (Lag 22 day)	22.38	NO_2_ (Lag 20 day)	21.12	NO_2_ (Lag 20 day)	17.12
NO_2_ (Lag 20 day)	12.38	O_3_ (Lag 22 day)	18.40	O_3_ (Lag 22 day)	4.81
NO (Lag 23 day)	7.29	NO (Lag 23 day)	7.47	CO (Lag 11 day)	3.81
Dew point (Lag 22 day)	7.01	Wind speed (Lag 19 day)	5.90	NO (Lag 23 day)	3.59
Wind speed (Lag 27 day)	5.71	Temperature (Lag 25 day)	4.78	Temperature (Lag 15 day)	2.99
CO (Lag 27 day)	5.31	Dew point (Lag 22 day)	4.37	PM2.5 (Lag 8 day)	2.90
SO_2_ (Lag 12 day)	3.41	Humidity (Lag 24 day)	4.07	SO_2_ (Lag 20 day)	2.12
Temperature (Lag 29 day)	2.71	CO (Lag 8 day)	3.50	Dew point (Lag 7 day)	1.90
PM2.5 (Lag 9 day)	2.46	PM2.5 (Lag 8 day)	3.25	Humidity (Lag 14 day)	1.57
Humidity (Lag 23 day)	1.91	SO_2_ (Lag 3 day)	2.90	Wind speed (Lag 8 day)	1.47
Pressure (Lag 24 day)	0.59	Pressure (Lag 4 day)	0.88	Pressure (Lag 20 day)	1.03

*Note*. COVID-19: Coronavirus disease 19; NO_2_: Nitrogen dioxide; O_3_: Ozone; NO: Nitric oxide; SO_2_: Sulfur dioxide; CO: Carbon monoxide; PM: Particulate matter; XGBoost: Extreme gradient boosting.

 The most important features affecting COVID-19 infection transmission based on the three techniques were NO_2_ at lag 5 day, CO at lag 4 day, T at lag 25 day, and O_3_ at lag 10 day (Table 2). Further, based on the majority voting with full prediction agreement of all the considered 3 models (100%), NO_2_ was reported to be the most important influencing factor in the transmission of the COVID-19 infection. The performance of DT, RF, and XGBoost predictive models for the COVID-19 infection was R^2^ = 0.56, R^2^ = 0.64, and R^2^ = 0.73, respectively (Table 2).

 The most important features affecting the severity of the disease and hospitalization based on the three ML techniques were RH at a lag of 28 days, T at a lag of 11 days, and O_3_ at a lag of 10 days (Table 3). Furthermore, based on the majority voting with moderate prediction agreement (2 out of 3 models, 66.7%), humidity was the most effective factor related to the severity of COVID-19. The performance of DT, RF, and XGBoost predictive models for hospitalizations due to COVID-19 was R^2^ = 0.68, R^2^ = 0.79, and R^2^ = 0.87, respectively (Table 3).

 Moreover, the most important features affecting mortality due to COVID-19 found by the three ML techniques were NO_2_ at lag 20 day, O_3_ at lag 22 day, and NO at lag 23 day (Table 4). Based on the majority voting with moderate prediction agreement (2 out of 3 models, 66.7%), NO_2_ was the most important factor affecting mortality related to COVID-19. The performance of DT, RF, and XGBoost predictive models for the number of deathsdue to COVID-19 was R^2^ = 0.53, R^2^ = 0.63, and R^2^ = 0.72, respectively (Table 4).

## Discussion

 Despite the large bodies of research on COVID-19, there are still huge gaps in understanding the mechanisms of infection transmission and the development of the disease. Various studies have shown that air pollution can increase the transmissibility and severity of several airborne infections, including coronavirus.^15,30^ The present study was conducted in Shiraz, the fourth largest city in Iran, to estimate the importance of major air pollutants (NO_2_, NO, O_3_, SO_2_, CO, and PM2.5) and meteorological parameters (AT, AH, WS, DP, and AP) on the pattern of case detection, hospitalization, and death due to COVID-19 using the ML techniques.

 According to the results, XGBoost provided the highest performance when compared to DT and RF. Positive and significant relationships were found between exposure to NO_2_, O_3_, PM2.5, AT, WS, and DP at particular lag s with the number of cases of COVID-19 and hospitalization. However, inverse associations were observed between NO, CO, RH, and AP levels and the number of new cases of COVID-19 and hospitalization. In addition, positive associations were found between NO_2_, O_3_, PM2.5, AT, WS, DP, and CO with the number of deaths caused by COVID-19. On the other hand, there was an inverse relationship between NO, RH, and AP and the number of deaths caused by COVID-19.

 Regarding the contribution of NO_2_ to the risk of COVID-19 infection, hospitalization, and mortality, the results of a study by Frontera et al also reported that NO_2_ may play an important role in the occurrence of severe forms of COVID-19.^31^. NO_2_ is released into the environment due to incomplete combustion in fuel engines and any process involved in burning coal, oil, or natural gas.^32^ In general, exposure to this pollutant is associated with an increased risk of mortality from respiratory and cardiac diseases. NO_2_ also causes alterations in the respiratory and immune functions, compromises the airflow in the airways, and ultimately facilitates complications of respiratory infections.^15^. Similarly, other researchers found a significant association between NO_2_ levels in the air and mortality from COVID-19. For example, a study conducted in 12 cities in Iran reported a relationship between air pollution (including NO_2_) and death due to COVID-19.^33^ Another study suggested that exposure to NO_2_ is more effective in increasing the mortality caused by COVID-19 compared to other air pollutants.^34^ In another study that was performed in 3 cities in Iran, NO_2_ was significantly associated with the mortality and severity of the COVID-19 infection.^6^ Regarding other pollutants, a study in India demonstrated, in addition to NO_2_, a significant rise in the number of COVID-19 cases following short-term exposure to PM2.5 and PM10.^35^ Similarly, the findings of another study revealed that exposure to PM2.5 was significantly associated with an increased risk of COVID-19 infection. It was also found that exposure to PM2.5, PM10, SO_2_, NO_2_, O_3_, and CO were associated with death due to COVID-19.^36^ In addition, a study conducted in Arak, Iran, indicated a positive and significant relationship between PM2.5, PM10, and SO_2 _and detected cases and deaths caused by COVID-19. In addition, air pollutants, including NO_2_, CO, O_3_, WS, and RH, were inversely associated with hospitalization and mortality of COVID-19.^37^ According to the WHO, the O_3_ level in the atmosphere causes respiratory problems and asthma and reduces lung function.^38^ In a study performed in India, PM2.5, PM10, SO_2_, and O_3_ were positively associated with the number of infections and deaths due to COVID-19.^38^ Likewise, research in China showed that the number of COVID-19 cases was positively correlated with the level of PM2.5, NO_2_, O_3_, and SO_2 _pollutants.^39^

 Several studies reported an association between hospitalization and exposure to SO_2_.^37^ Similarly, Liu et al in China demonstrated that the rate of spread of the COVID-19 infection increased with exposure to SO_2_.^40^ However, Zhu et al found no association between exposure to SO_2 _and hospitalization.^39^

 It seems that SARS-CoV-2 can spread wider in windy conditions because it increases air circulation.^41^ In the present study, a direct relationship was observed between average WS and mortality due to COVID-19. A study conducted in Malaysia revealed that the cases of COVID-19 were negatively correlated with WS.^24^ Air PMs, especially PM2.5, may not only carry SARS-CoV-2 but also promote virus attachment and replication in the bronchi by damaging bronchial epithelial cells, ultimately leading to increased hospitalizations and death due to COVID-19.^42,43^

 Several studies evaluated the role of meteorological parameters in the transmission of COVID-19.^44,45^ Accordingly, different environmental conditions were found to affect the spread of the infection by changing several parameters, including air conditioning and circulation.^45^ Air T and humidity were the most important meteorological parameters affecting mortality caused by COVID-19. Air T could affect the spread of COVID-19 under different conditions. For example, high RH was reported to have an inhibitory effect on the transmission of COVID-19.^46^ For instance, according to a study performed in Ahvaz, high air T and high RH led to a significant decrease in the daily incidence of COVID-19.^13^ Another study in the UK found that high T and long DPs could reduce the incidence of COVID-19 and its related mortality. On the other hand, WS significantly increased the above indexes.^41^ In line with the results of the present study, some studies showed a positive and significant correlation between air T and the number of patients with COVID-19.^47^ This is due to the hot weather outside, which forces people to stay in closed environments without ventilation, and the risk of disease transmission increases as a result of insufficient ventilation in indoor spaces.^48,49^ However, several studies reported an inverse correlation between air T and the spread and mortality of coronavirus.^50^. It is also possible that due to sunlight and UV radiation in the seasons with high T, environmental conditions are tougher for any infectious agent in the air, including COVID-19.^24,37,41^

 Regarding the studies discussed above, no lag time was investigated when investigating the effect of air pollutants and meteorological factors on COVID-19 infection and mortality. At the current point, we are unable to interpret the lag s between exposure to the air and the COVID-19 infection. However, we believe that defining the lag time with an interval of several days compared to the one-day model provides a better understanding of the effects of air pollution on airborne infections, including COVID-19.

## Strengths and Limitations

 A range of major meteorological and air quality indexes were used to better understand the association and temporality between these factors and COVID-19 infection incidence and severity. However, the nature of the study design prevents us from making causal inferences from the results, as many unmeasured factors (e.g., fast-changing and hard-to-track behavioral factors in the study population regarding the transmission of infection, detection of infection, and hospitalization) are potentially confounding the associations under study.

**Highlights**

- NO_2_ (lag 5 day), CO (lag 4), and temperature (lag 25) are the most important environmental features affecting the spread of the COVID-19 infection.
- The most important features affecting hospitalization due to COVID-19 are RH (lag 28), temperature (lag 11), and O_3_ (lag 10).
- The most essential features affecting the number of deaths caused by COVID-19 are NO_2_ (lag 20), O_3_ (lag 22), and NO (lag 23).
- Air pollutants and meteorological parameters can be used to predict the disease burden of COVID-19 on the community and health system.

## Conclusions

 In this study, several air pollutants and meteorological parameters were analyzed to measure the correlation between NO_2_, NO, O_3_, SO_2_, PM2.5, CO, air T, RH, WS, DP, and AP and the transmission and severity of the COVID-19 infection in Shiraz. The findings confirmed positive and strong (*r* > 0.5) associations between NO_2 _(lag 8 days) and O_3_ (lag 6 days) with case detection, and NO_2_ (lag 20 days) and O_3_ (lag 23 days) with mortality of COVID-19 cases. Humidity (lag 26 and lag 30 days) and pressure (lag 30 and lag 29 days) were negatively and significantly associated with both case detection and death due to COVID-19. Our results suggested that air pollutants and meteorological parameters with particular lag times play important roles in the epidemiology of the disease. Thus, it is necessary to perform further studies to understand the mechanism of the delayed effect of such pollutants on the natural history of the COVID-19 infection. Regarding the application of the results, the air quality and condition parameters should be used when studying such airborne epidemics and should be considered when developing and implementing primary and secondary preventive strategies. Specific measures, such as traffic management and specifically targeted partial community or social event lockdowns, can be taken to reduce air pollution and infection transmission in communities. Meteorological and air quality data can be integrated into public health policies to mitigate the impact of COVID-19 and other respiratory diseases.

## Acknowledgments

 We highly appreciate the contribution of the staff at the Medical Care Monitoring Center of Shiraz University of Medical Sciences to providing COVID-19 data. We also thank the Environmental Organization of Fars Province for kindly providing us with the air pollution data.

## Authors’ Contribution

**Conceptualization: **Nadia Mohammadi Dashtaki, Alireza Mirahmadizadeh, Mohammad Fararouei, Mohammad Hoseini.

**Data curation:** Nadia Mohammadi Dashtaki, Alireza Mirahmadizadeh, Mohammad Reza Nayeb.

**Formal analysis: **Nadia Mohammadi Dashtaki.

**Funding acquisition: **Mohammad Fararouei.

**Investigation: **Nadia Mohammadi Dashtaki.

**Methodology: **Nadia Mohammadi Dashtaki, Reza Mohammadi Dashtaki.

**Project administration: **Mohammad Fararouei.

**Resources: **Mohammad Fararouei.

**Software:** Nadia Mohammadi Dashtaki, Reza Mohammadi Dashtaki.

**Supervision: **Mohammad Fararouei.

**Validation: **Mohammad Fararouei.

**Visualization**: Nadia Mohammadi Dashtaki.

**Writing–original draft: **Nadia Mohammadi Dashtaki.

**Writing–review & editing:** Mohammad Fararouei, Nadia Mohammadi Dashtaki, Alireza Mirahmadizadeh.

## Competing Interests

 The authors declare that they have no competing interests.

## Ethical Approval

 This study was approved by the Local Ethics Committee of Shiraz University of Medical Sciences (SUMS) by code No. IR.SUMS.SCHEANUT.REC.1402.108.

## Funding

 None.
